# The complete mitochondrial genome of *Sardinops sagax* (Jenyns, 1842) (Clupeiformes: Clupeidae) and phylogenetic analyses of sardines

**DOI:** 10.1080/23802359.2021.1899867

**Published:** 2021-03-18

**Authors:** Fenghua Tang, Weitao Chen

**Affiliations:** a Key Laboratory of Oceanic and Polar Fisheries, Ministry of Agriculture and Rural Affairs; East China Sea Fisheries Research Institute, Chinese Academy of Fishery Sciences, Shanghai, China; b Pearl River Fisheries Research Institute, Chinese Academy of Fishery Science, Guangzhou, China; c Experimental Station for Scientific Observation on Fishery Resources and Environment in the Middle and Lower Reaches of Pearl River, Zhaoqing, China

**Keywords:** *Sardinops sagax*, mitogenome, sardine species, phylogeny

## Abstract

Pacific sardine *Sardinops sagax* (Jenyns, 1842), a sardine species that widely distributes in Pacific, is an important commercial species in many areas. In this study, we characterized the complete mitochondrial genome of *S. sagax* using next generation sequencing technology. The complete mitogenome of *S. sagax* was 16,883 base pairs (bp) in length and comprised 13 protein-coding genes (PCGs), 22 transfer RNA (tRNA) genes, 2 ribosomal RNA genes (rRNA), and one control region (D-loop). Phylogenetic analysis indicated that sardine species included three clades (I, II and III) and *S. sagax* clustered with *Sardinops melanostictus*.

Pacific sardine *Sardinops sagax* (Jenyns, 1842) is an important commercial species that supported an important fishery in Pacific waters (www.fishbase.org), which has wide distributions from southern Africa to eastern Pacific. Currently, this species occupies 10%–40% of fishery production in the Northwest Pacific. Empirical data argued that the resources of Pacific sardine had undergone long-term fluctuations in history, which were influenced by climate and current (Yatsu et al. 2005). In this study, we sequenced and characterized the complete mitochondrial genome of *S. sagax* from the Pacific Northwest. The mitogenome can provide basic genetic information for subsequent population genetic and phylogenetic studies.

A *S. sagax* individual was sampled from the Northwest Pacific (36.4251 N, 158.6026E) in May 2020. This sample (Voucher number: YDNSD20200501) was stored in the fish collection of East China Sea Fisheries Research Institute. Total genomic DNA was extracted from a bit of tail fin using a Genomic DNA Isolation Kit (QiaGene, Germany). The complete mitochondrial genome was sequenced using the Illumina MiSeq platform (Illumina Inc, San Diego, CA, USA). Finally, we *de novo* assembled the complete mitochondrial genome using SPAdes 3.9.0 (Bankevich et al. 2012) and annotated Protein-coding genes, rRNA genes and tRNA genes using MITOS (Bernt et al. 2013).

The complete mitochondrial genome of *S. sagax* was 16,883 base pairs (bp) in length and comprised of 13 protein-coding genes (*ND1*, *ND2*, *ND3*, *ND4*, *ND4L*, *ND5*, *ND6*, *COI*, *COII*, *COIII*, *ATP6*, *ATP8*, *Cyt b*), 2 rRNA genes (12S rRNA and 16S rRNA), 22 tRNA genes and a control region (D-loop) (GenBank nos: MW338734). Its structural organization and gene order were similar with those in other typical teleosts. A total of 17 sardine mitogenomes including 16 published mitogenomes were aligned using MUSCLE (Edgar 2004). We choose 13 protein-coding genes manually and then were combined into a single sequence for phylogenetic analysis. Maximum-likelihood tree was implemented in RAXML-VI-HPC (Stamatakis 2006) using a GTR + I + G model. Nodal support values were estimated from 1000 nonparametric bootstrap replicates.

The maximum-likelihood tree obtained three well supported major clades with high supported values (Clades I, II and III; Figure 1). Clade I contained species from five genera (*Clupea*, *Ethmidium*, *Nematalosa*, *Sardinella* and *Sardinops*), Clade II comprised *Engraulis* species and Clade III consisted of *Hyperlophus vittatus*. Furthermore, *S. sagax* was clustered with *Sardinops melanostictus* (Figure 1), suggesting these two species had a closely related phylogenetic relationship.

**Figure 1.**
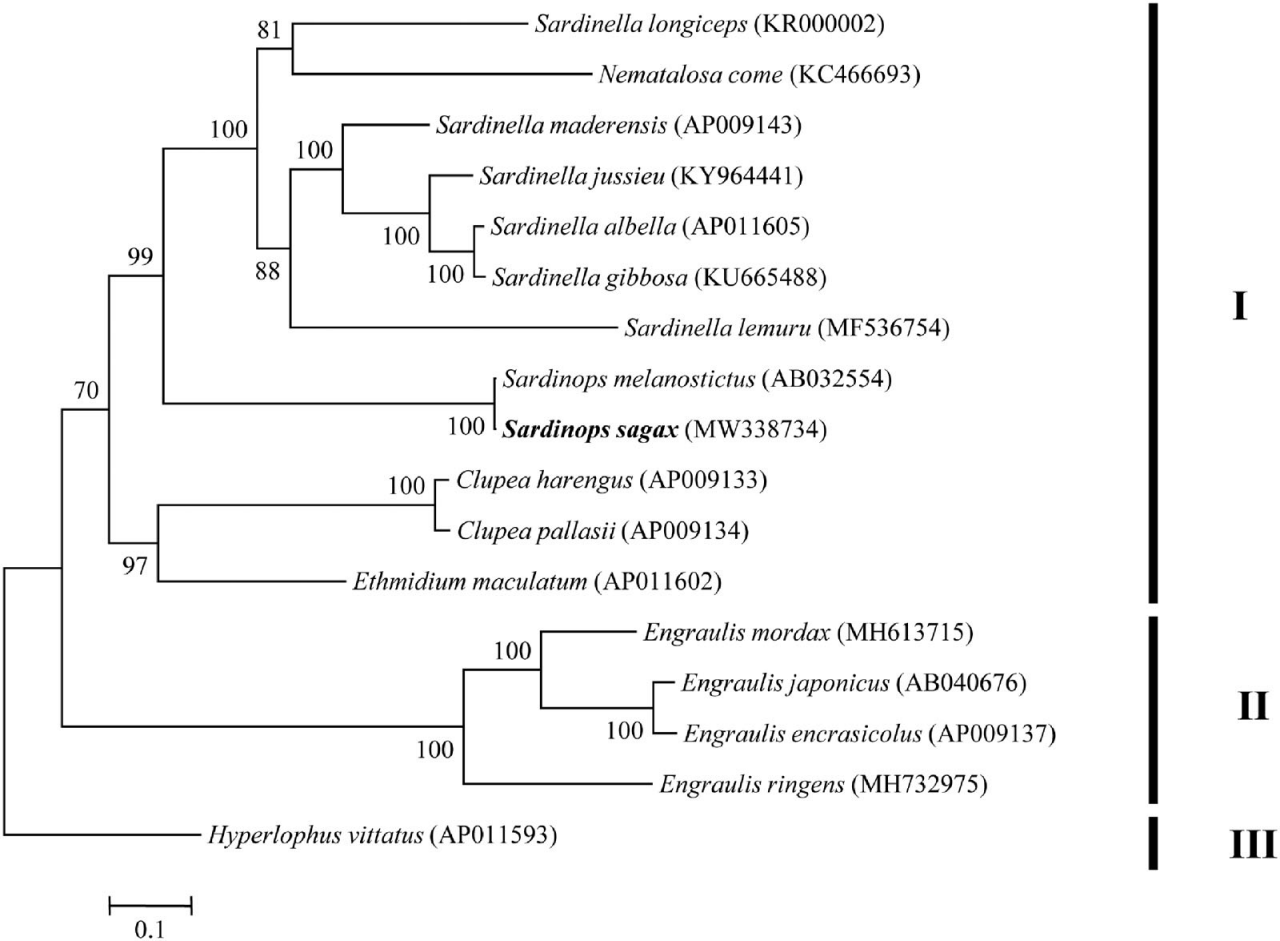
Maximum-likelihood tree showing the phylogenetic relationships among 17 sardine mitogenomes based on 13 protein-coding genes. Values on branches indicate bootstrap values from maximum-likelihood analysis.

## Ethical approval

Experiments were performed in accordance with the recommendations of the Ethics Committee of East China Sea Fisheries Research Institute. These policies were enacted according to the Chinese Association for the Laboratory Animal Sciences and the Institutional Animal Care and Use Committee (IACUC) protocols.

## Data Availability

The data that support the findings of this study is openly available in GenBank of NCBI at http://www.ncbi.nlm.nih.gov, reference number MW338734. The associated BioProject, SRA, and Bio-Sample numbers are PRJNA695793, SRR13575827 and SAMN17620116, respectively.
